# Identification of noninvasive diagnostic biomarkers for ectopic pregnancy using data-independent acquisition (DIA)proteomics: a pilot study

**DOI:** 10.1038/s41598-022-23374-8

**Published:** 2022-11-21

**Authors:** Dan Ma, Ruiqing Yang, Yunlong Chen, Zhengyi Huang, Yuxin Shen, Chengqi He, Lixing Zhao

**Affiliations:** 1 Department of Rehabilitation Medicine, West China Second University Hospital, Sichuan University, Chengdu, Sichuan China; 2 West China Medical School of Sichuan University, Chengdu, Sichuan China; 3 Key Laboratory of Birth Defects and Related Diseases of Women and Children (Sichuan University), Ministry of Education, Chengdu, Sichuan China; 4 Department of Rehabilitation Medicine Center, West China Hospital, Sichuan University, Chengdu, Sichuan China; 5 Key Laboratory of Rehabilitation Medicine in Sichuan Province, Chengdu, China; 6 State Key Laboratory of Oral Diseases, National Clinical Research Center for Oral Diseases, Chengdu, Sichuan China; 7 Department of Orthodontics, West China Hospital of Stomatology, Sichuan University, Chengdu, Sichuan China

**Keywords:** Diagnostic markers, Biomarkers

## Abstract

At present, the diagnosis of ectopic pregnancy mainly depends on transvaginal ultrasound and β-hCG. However, these methods may delay diagnosis and treatment time. Therefore, we aimed to screen for serological molecular markers for the early diagnosis of ectopic pregnancy (EP).Using data-independent acquisition (DIA)proteomics, the differential proteins in serum were selected between the intrauterine pregnancy (IP) and EP groups. Then, the expression levels of these differential proteins were measured by enzyme-linked immunosorbent assay. The diagnostic value of the serum biomarkers was evaluated by receiver operating characteristic curve analysis.GSTO1, ECM-1 and β-hCG showed significant differences between the EP and IP groups (*P* < 0.05). The combination of GSTO1/ECM-1/β-hCG had an area under the curve of 0.93 (95% CI 0.88–0.99), a sensitivity of 88.89% (95% CI 73.94–96.89) and a specificity of 86.11% (95% CI 70.50–95.33) with a likelihood ratio of 6.40.The combination of GSTO1/ECM-1/β-hCG may be developed into a possible approach for the early diagnosis of EP.

## Introduction

Ectopic pregnancy(EP) is defined as a pregnancy that occurs outside the uterus, most commonly in a fallopian tube,it can also occur in an ovary, the abdominal cavity, and a uterine horn^1^. The prevalence of ectopic pregnancy is estimated at 2% of all pregnancies, is a significant cause of mortality in pregnant women^2^. At present, it still accounts for 6% of pregnancy-associated morbidities^3^. Typical symptoms of ectopic pregnancy are cessation of menstruation, sudden abdominal pain, and vaginal bleeding^4^.In severe cases, syncope, shock and even death may occur^4^. Therefore, the diagnosis and early treatment of ectopic pregnancy are especially important. At present, the diagnosis of ectopic pregnancy mainly depends on transvaginal ultrasound and the quantitative measurement of the β subunit of human chorionic gonadotropin (β-hCG)^5^. However, in practice, a patient only visits the doctor after abdominal pain and vaginal bleeding, which easily delays the diagnosis and treatment time. Moreover, current diagnostic methods have to be done in the hospital, we want to develop an early screening method for ectopic pregnancies that can be used at home,so as to greatly reduce the harm of ectopic pregnancy.Therefore, early warning and better diagnosis of ectopic pregnancy are urgent issues.

In the last 20 years, researchers have been searching for serological markers of ectopic pregnancy, but little progress has been made thus far due to various difficulties. At present, β-hCG is still the most widely used serum marker in clinical practice, but a single serum β-hCG level can only reflect pregnancy but not the pregnancy location. Therefore, serum β-hCG is not suitable for the diagnosis of ectopic pregnancy.

To better diagnose ectopic pregnancy, several studies have been carried out, and several indicators have been identified, such as progesterone, VEGF, inhibin A, activin A, or a combination of progesterone, β-hCG, CA125, and CD3 + T cell percentages^6^. In 2007, Florio et al. first reported that activin A can be used to predict ectopic pregnancy^7^. When the detection limit was set at 0.37 ng/mL, the sensitivity and specificity were 100% and 99.6%, respectively. However, the result was not confirmed in subsequent confirmatory studies. Yan et al. reported that the sensitivity and specificity of Adrenomedullin (ADM) in the detection of ectopic pregnancy were only 53.50% and 85.00%,respectively^8^. However, these factors are limited in practicability due to conflicting results or low sensitivity and specificity.

Data Independent Acquisition (DIA) is an emerging quantitative proteomics technique enabling fast and sensitive protein profiling from complex mixtures. This detection provides an unambiguous identification and quantification of all the lipids (both low and high abundant peaks).Utilizing novel scan functions and data processing workflows DIA represents a paradigm shift in the expectations associated with quantitative proteomics analysis^9^. The aim of this study was to screen for serological molecular markers of ectopic pregnancy with the application of data-independent acquisition (DIA)-based quantitative proteomics.

## Results

### Clinical findings

In this study, we recruited 36 women with ectopic pregnancy and 36 women with normal early pregnancy as controls after clinical evaluation, β-hCG and vaginal ultrasound examination. Demographic information and clinical characteristics are given in Table 1. Pregnancy outcomes included 36 (50.0%) viable IPs and 36 (50.0%) EPs. Twenty-seven EPs (75.0%) were placed in fallopian tubes, and nine (25.0%) were placed in uterine incisions. Both groups of women were in the early stages of pregnancy, and no significant difference was observed in terms of age and gestational age (*P* > 0.05), which was determined by the first day of the last menstrual period. In addition, β-hCG levels were measurable in all samples and were significantly (*P* < 0.001) lower in the EP group than in the IP group (Table 1).

**Table 1:** Baseline of the study population.

	IP (n = 36)	EP (n = 36)	*P* value
Age, year (± SD)	29.50 ± 5.01	30.33 ± 4.77	0.47
Gestational age, day (± SD)	52.19 ± 8.25	51.36 ± 10.94	0.72
β-hCG (mIU/mL)	75,939.89 ± 61,751.00	18,636.28 ± 28,190.06	< 0.001
Site of EP (%)			
Tubal Ectopic Pregnancy(TEP)		27 (75.0)	
Uterine incision		9 (25.0)	

*IP* intrauterine pregnancy, *EP* ectopic pregnancy, *SD* standard deviation, *hCG* human chorionic gonadotropin.

### Molecular markers screening

**Figure 1:**
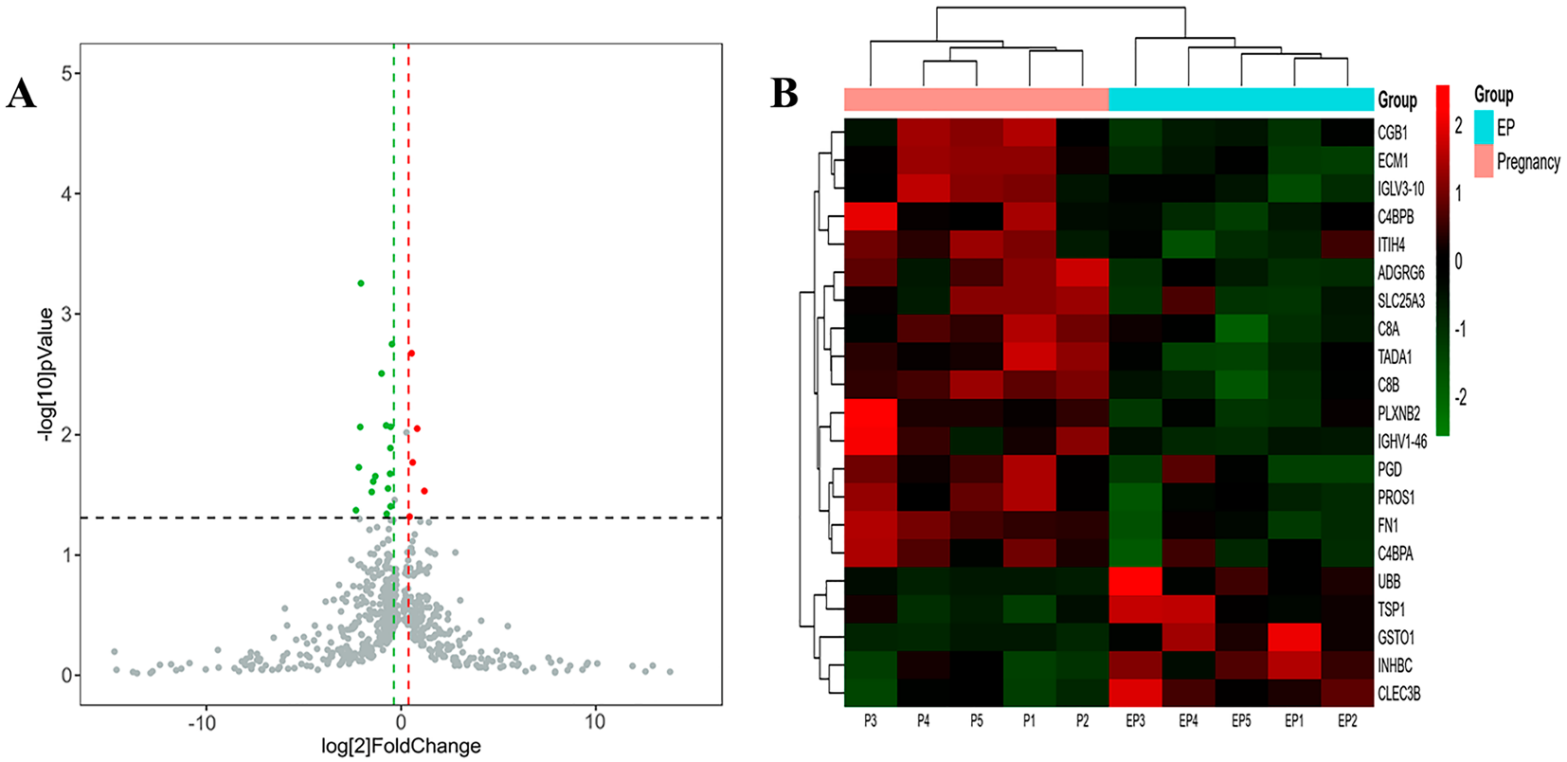
Volcano plot (**A**) and Cluster heatmap (**B**) of the differentially expressed proteins between IP and EP group.

Potential biomarkers in the serum samples for evaluating activity were screened using data-independent acquisition (DIA)proteomics. As shown in Fig. 1, in the 5 samples evaluated, each sample has multiple positive points. The intensities of TSP1, GSTO1, INHBC, CLEC3B and ECM-1 in the serum samples of the EP group were significantly different from those in the IP group. These results indicate that these serological markers may be useful targets for the diagnosis of EP.

### Molecular markers confirmation

**Figure 2:**
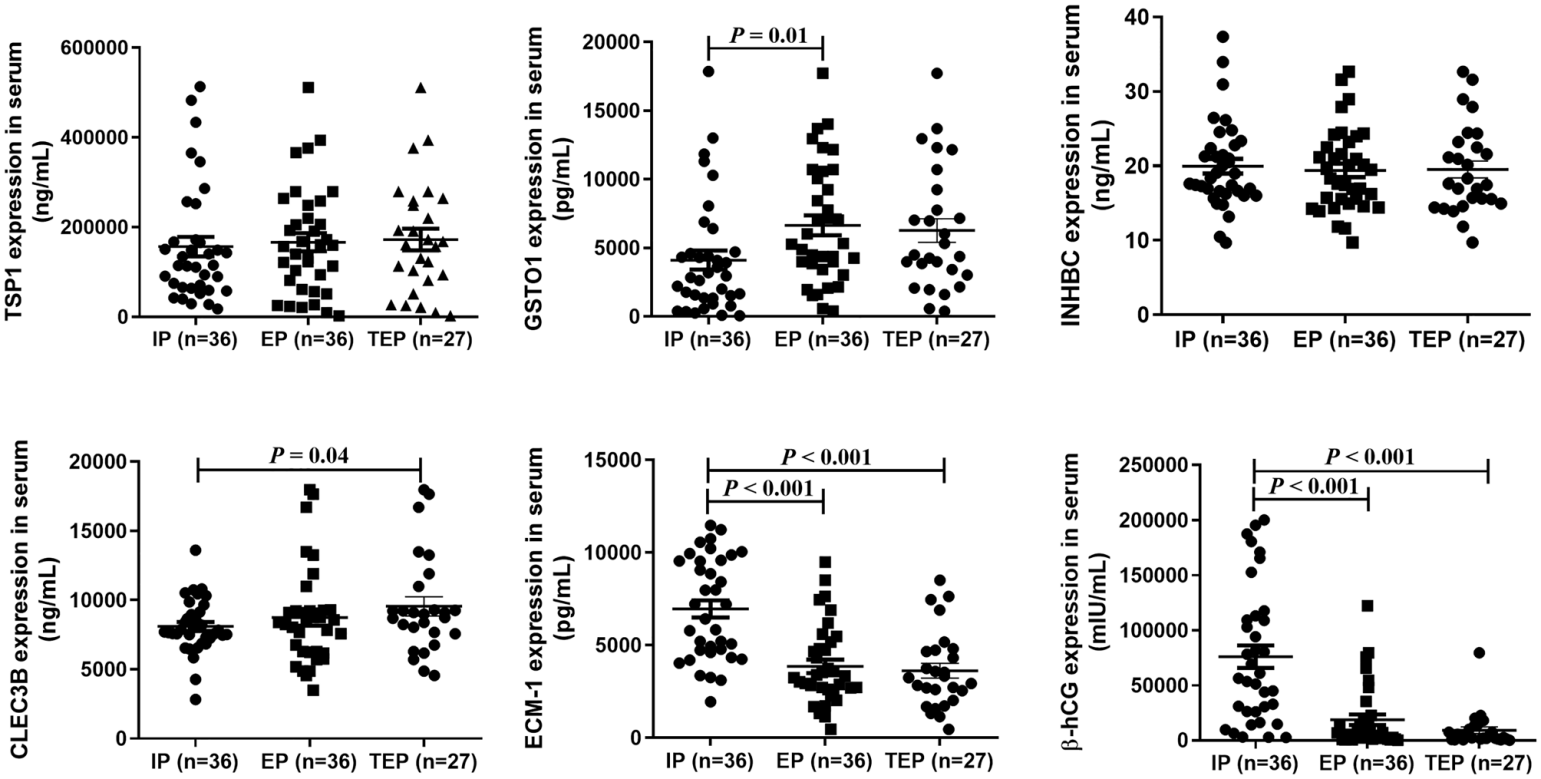
Concentrations of TSP1, GSTO1, INHBC, CLEC3B, ECM-1 and β-hCG in serum of IP and EP women. Data are presented as the mean ± standard deviation.

The expression of TSP1, GSTO1, INHBC, CLEC3B, ECM-1 and β-hCG in serum was measured by using ELISA (n = 36). As shown in Fig. 2, the expression levels of GSTO1 were significantly higher in patients with EP than in patients with normal pregnancy (*P* = 0.01). Although CLEC3B showed no difference between EP and IP, the expression level of CLEC3B in TEP decreased significantly (*P* = 0.04).Interestingly, ECM-1 concentrations in EP and TEP were significantly lower than those in IP (*P* < 0.001). The β-hCG levels were significantly lower in EP and TEP pregnancies than in normal pregnancies (*P* < 0.001). There was no statistically significant difference in TSP1, INHBC or CLEC3B between the three groups.

**Figure 3:**
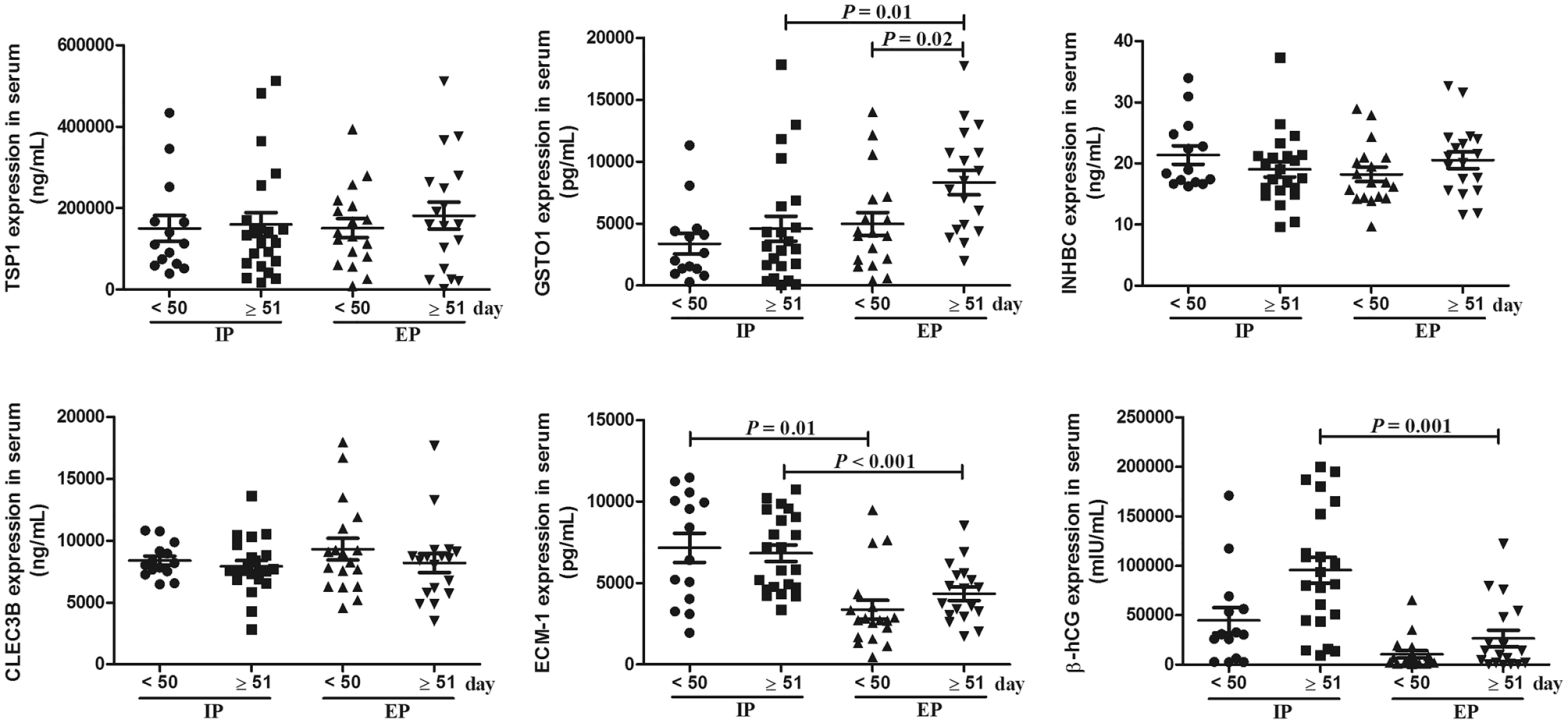
Subgroup analysis of concentrations of TSP1, GSTO1, INHBC, CLEC3B, ECM-1 and β-hCG in serum of IP and EP women according to gestational age (< 50 vs. 51 days). Data are presented as the mean ± standard deviation.

Subgroup analysis according to gestational age (< 50 vs. ≥ 51 days) showed that the GSTO1 levels were significantly higher in patients with EP whose gestational age ≥ 51 days compared to patients with IP whose gestational age ≥ 51 days (*P* = 0.01) and patients with EP whose gestational age < 50 days (*P* = 0.02). ECM-1 concentrations in EP patients whose gestational age was < 50 days were significantly lower than those in IP patients (*P* = 0.01). The same lower levels of ECM-1 were also observed in EP patients whose gestational age was ≥ 51 days (*P* < 0.001). Moreover, the β-hCG concentrations of EP patients whose gestational age ≥ 51 days were lower than those of IP patients whose gestational age ≥ 51 days (*P* = 0.001) (Fig. 3).

### Diagnostic accuracy of biomarkers for EP

**Figure 4:**
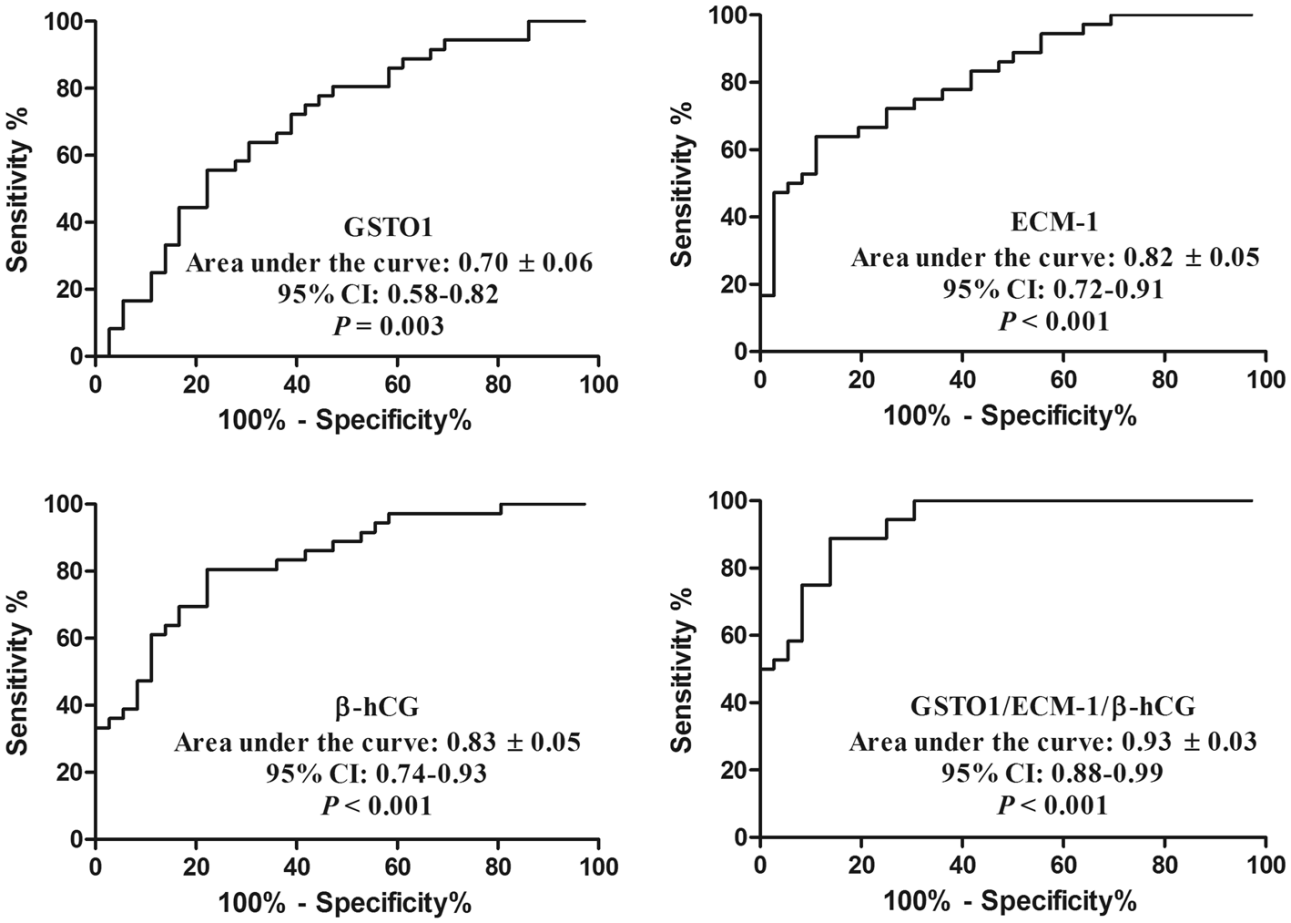
Receiver operating characteristic curves of GSTO1, ECM-1 and β-hCG.

The ROC curve was used to evaluate the sensitivity/specificity, positive/negative predictive value, likelihood ratio (LR) and area under the curve (AUC) of GSTO1, INHBC, β-hCG and different combinations as diagnostic tests. Table 2 and Fig. 4 demonstrate the significant discriminatory ability of increased GSTO1, ECM-1, β-hCG and different combination levels for the diagnosis of EP.

**Table 2:** Diagnostic value of GSTO1, ECM-1 and β-hCG for EP.

	Cut off	Sensitivity (95% CI), %	Specificity (95% CI), %	Likelihood ratio
GSTO1 (pg/mL)	4343	63.89 (46.22–79.18)	69.44 (51.89–83.65)	2.09
ECM-1 (pg/mL)	4732	72.22 (54.81–85.80)	75.00 (57.80–87.88)	2.89
β-hCG (mIU/mL)	24,300	80.56 (63.98–91.81)	77.78 (60.85–89.88)	3.63
GSTO1/ECM-1	0.3296	88.89 (73.94–96.89)	72.22 (54.81–85.80)	3.20
GSTO1/ECM-1/β-hCG	− 2.094	88.89 (73.94–96.89)	86.11 (70.50–95.33)	6.40

*GSTO1* glutathione S transferase omega 1, *ECM-1* extracellular matrix protein-1, *hCG* human chorionic gonadotropin, *EP* ectopic pregnancy, *CI* confidence interval.

In detail, GSTO1 at the cutoff of 4343 pg/mL achieved a sensitivity of 63.89% (95% confidence interval (CI), 46.22–79.18) and a specificity of 69.44% (95% CI, 51.89–83.65) as a single serum marker for EP prediction (AUC: 0.70 (95% CI, 0.58–0.82)), with an LR of 2.09. When an ECM-1 concentration of 4732 pg/mL was used as a cutoff for the diagnosis of EP in the control group, the sensitivity was 72.22% (95% CI, 54.81–85.80), the specificity was 75.00% (95% CI, 57.80–87.88) as a single serum marker for EP prediction (AUC: 0.82 (95% CI, 0.72–0.91)), and the LR was 2.89. β-hCG at the cutoff of 24,300 mIU/mL achieved a sensitivity of 80.56% (95% CI, 63.98–91.81) and a specificity of 77.78% (95% CI, 60.85–89.88). The AUC for β-hCG was 0.83 (95% CI: 0.74–0.93), with an LR of 3.63. When combining all the factors together, we constructed the Formula y = (0.0003*GSTO1)-(0.001*ECM1)-(0.00003*hCG) + 3.518. The combined detection of these three serological markers had an AUC of 0.93 (95% CI, 0.88–0.99), a sensitivity of 88.89% (95% CI, 73.94–96.89) and a specificity of 86.11% (95% CI, 70.50–95.33) with an LR of 6.40.

## Discussion

Given that diagnosing ectopic pregnancy is a clinical challenge in the early gestational age, how to explore a biomarker to simplify and improve the diagnosis of ectopic pregnancy is a research focus.Some studies demonstrated that there is no single diagnostic biomarker for tubal ectopic pregnancy that has been adequately tested and yields satisfactory results^10^. Using multiple serum marker analysis might be a possible solution for the diagnosis of EP. In this study, we found that GSTO1, ECM-1 and β-hCG may be serological markers for the early diagnosis of EP, especially when combining the three factors together, with the best sensitivity and specificity.

Every marker that we choose is biologically plausible.Glutathione transferase (GST) is a large family of transferases that is related to the progression of tumors and the metabolism of foreign objects (such as environmental pollutants)^11^. Omega-GST 1 has been confirmed to exist widely in a variety of tissues and has the activity of a variety of biological enzymes, especially those involved in the biotransformation of arsenic^12–14^. Some studies have found that there is a statistical relationship between recurrent abortion and GSTO1 mutation during pregnancy. They further speculated that this relationship is related to the change in ascorbate reductase activity and arsenic metabolism caused by GSTO1 mutation^15^. In a study of the relationship between pregnancy and GSTO1, GSTO1 was also found to be related to fetal intrauterine growth restriction^16,17^. In this study, we found that for more than 51 days of pregnancy, GSTO1 levels in the IP and EP groups were significantly different, but the sensitivity and specificity of the difference were not high.

Extracellular matrix protein 1 (ECM-1) is a glycoprotein that usually acts as a functional binding core protein and interacts with a variety of proteins to regulate angiogenesis and tumor growth^18^. For example, the interaction of ECM-1 with matrix metalloproteinase 9 (MMP9), the epidermal growth factor receptor (EGFR), CX3CL1 and CCL14 is involved in the occurrence and development of many diseases^19–21^. Graubner et al. proposed that a large amount of ECM1 can be observed in the endometrial epithelium at the early stage of pregnancy, which may be related to decidualization and implantation preparation^22^. As pregnancy progresses, the amount of ECM-1 changes accordingly, suggesting that ECM-1 plays an important role in maintaining pregnancy and fetal development^23,24^. Hannan et al. found that CX3CL1 and CCL14 promote trophoblast migration in early pregnancy by regulating ECM-1^20^. Therefore, we explored the relationship between ECM-1 and EP and found a significant difference between the IP group and EP group, suggesting the possibility of ECM-1 in the diagnosis of early EP.

Other markers of pregnancy development were less significant.Thrombospondin-1 (TSP-1) is a sensitive index for monitoring platelet activation *in vitro*^25^. Studies have shown that the expression of TSP-1 has a certain relationship with pregnancy and the expression level in different muscle tissues is different at different stages of pregnancy, which ensures the normal formation of blood vessels during pregnancy and maintains the stability of the blood vessel bed^26,27^. Inhibin beta C (INHBC) belongs to the transforming growth factor beta (TGF-β) family, widely exists in the placenta and endometrium, and has an anti-cell proliferation effect^28–30^. Abdoli et al. and Fortes et al. pointed out that the INHBC gene is involved in the inheritance of reproductive traits of sheep and cattle^31,32^, and some scholars also pointed out that INHBC is involved in the blastocyst implantation process^33^. C-type lectin domain family 3 member B (CLEC3B) is a binding protein that has a specific binding effect on plasminogen kringle-4^34^. Rocha et al. proposed that the fetus can stimulate CLEC3B secretion during pregnancy, so CLEC3B has a role in the diagnosis of early pregnancy^35^. Unfortunately, in this study, we did not find a significant difference in these three markers between the IP group and EP group, but further study is needed.

As the most commonly used serological indicator of EP, β-hCG plays a very important role in the diagnosis of early ectopic pregnancy. Often, low levels of β-hCG may suspect the possibility of EP, but β-hCG alone does not confirm or rule out EP, which was consistent with our experimental results^36–38^. Although there were significant differences between the IP group and the EP group, the sensitivity and specificity of β-hCG for the diagnosis of EP were poor, and GSTO-1 and ECM-1 showed the same results. However, when β-hCG, GSTO-1 and ECM-1 were combined, both sensitivity and specificity were significantly increased, suggesting the possibility of early diagnosis of EP.

Due to the limited success of single serum biomarker measurements, several researchers have started to investigate the possibility of using several markers analysis in order to diagnose tubal ectopic pregnancy.O'Leary et al. examined progesterone and β-hCG levels and found, in preliminary studies, that a plasma β-hCG < 3000 IU/l and a plasma progesterone < 40 nmol/l could predict a tubal ectopic pregnancy with a sensitivity of 88% and specificity of 82%^39^. A retrospective study analyzed 289 women in the emergency department who were diagnosed with EP, spontaneous abortion, or viable intrauterine pregnancy,and the researchers collected the serum progesterone, hCG and activin A concentrations of these patients and made statistical analysis^40^. Their findings suggest that progesterone (< 10 ng/ml), hCG (< 6,699 IU/l), and activin A (< 0.26 ng/ml) cutoffs were optimized by ROC analysis,and the multi-marker panel utilizing all three biomarkers had a sensitivity of 70% and specificity of 69%^40^. Our study also confirms these findings that combining several markers into a single test with better diagnostics than individual proteins.In addition, the results of our study were similar to those of the above studies in terms of sensitivity and specificity.

There are limitations in this experiment. First,these findings are considered preliminary and many of the analyses are not routinely used in clinical practice. External validation of future plans will be conducted in a separate set of samples. Second, our sample size is relatively small, which may lead to deviation of the results. It is suggested that a larger sample size should be used in future research. Moreover, the results of various proteins in the IP group and EP group showed little difference, which may not be applied to the clinical rapid examination index.

In conclusion, the present study reveals that GSTO1/ECM-1/β-hCG in serum may be potentially useful biomarkers for the early diagnosis of EP, which should be confirmed in studies with larger sample sizes.We hope to present this research result to provide inspiration for subsequent researchers in research design and research ideas.

## Methods

### Patients and serum sample collection

In the EP group, the inclusion criteria included 4–12 weeks of pregnancy, abdominal pain, bleeding and other clinical symptoms, and diagnosis of EP confirmed by transvaginal ultrasonography(including tubal pregnancy, uterine incision scar pregnancy and uterine horn pregnancy). Women with a history of menopause and increased serum β-hCG but without diagnosis confirmed by ultrasound or improvement after conservative treatment were excluded. In the intrauterine pregnancy (IP) group, the inclusion criteria included 4–12 weeks of pregnancy, no abdominal pain, bleeding or other clinical symptoms, and uterine fetal bud confirmed by ultrasound. Clinical data were recorded for all patients, including age, gestational age, serum β-hCG concentration, progesterone concentration, and clinical diagnosis.This study was approved by the Ethics Committees of West China Second Hospital of Sichuan University(no.124), and all patients signed informed consent forms.

2 to 3 ml of a peripheral venous blood sample of EP and IP patients was each collected in third-grade A hospitals. Samples were centrifuged at 1000 rpm at 4 °C for 10 min, and the serum was separated, packaged and stored at -80 °C until use. All samples were selected from unrelated Han individuals.

### Proteomic screening assay

Ten serum samples (from 5 EP patients and 5 IP patients) were used to perform proteomic screening by data-independent acquisition (DIA). All of the selected samples were matched for age and gestational age(see supplementary Table 1).

The first stage is sample extraction and quality control. First, 10 μl serum from each sample (containing trypsin inhibitors) was added to the resin slurry in the column at room temperature (RT). After mixing, the column was placed on the rotator and rotated for 1 h. Second, with the bottom and lid removed, the column was placed in a 2 ml EP tube and centrifuged at 1,000 g for 2 min at 4 °C. Third, after removal of the top 12 proteins in abundance from plasma, the eluted samples were reserved by vacuum freeze drying. The lyophilized sample was added to 100 μl SDS for redissolution and subsequently centrifuged at 12,000 g for 10 min at RT. The supernatant was the total protein solution, whose concentration was determined by the bicinchoninic acid (BCA) method. Finally, each sample (8 µg) was separated by 12% SDS polyacrylamide gel electrophoresis (SDS–PAGE). After that, the separated gels were stained by Coomassie brilliant blue staining and subsequently washed with distilled water until the background was clear. For quality control, the gels were scanned by using ImageScanner, which was used to judge whether follow-up experiments were possible according to the images.

The second stage is DIA experiment. First, after protein quantification, a 30 μg sample was placed in an ultrafiltration tube and reacted with 120 μl reductant buffer (10 mM DTT, 8 M urea, 100 mM TEAB; pH 8.0) at 60 °C for 1 h. Following the reaction, IAA was added until the final concentration was 50 mM, and the reaction time was 40 min at RT without light. The solution at the bottom of the collecting tube was discarded after centrifugation at 12,000 rpm at 4 °C for 20 min. After that, 100 μl buffer (300 mM TEAB) was added to the tube and centrifuged twice at 12,000 rpm for 20 min. After replacement of the collection tube, 100 μl buffer (300 mM TEAB) and 2 μl sequencing grade trypsin solution (1 μg/μl) were added to the ultrafiltration tube and reacted at 37 °C for 12 h. The peptides after enzymolysis were collected after centrifugation at 12,000 rpm for 20 min. Subsequently, 50 μl buffer (200 mM TEAB) was added to the tube and centrifuged at 12,000 rpm for 20 min. The solution at the bottom of the tube was collected and lyophilized. Second, after enzymolysis and lyophilization, the peptides were redissolved with 1 ml 0.1% TFA and desalted with an RP-C18 solid phase extraction (SPE) column. Third, for LC–MS/MS analysis, the IRT standard sample and the sample to be tested were mixed according to a volume ratio of 1:10, and mass spectrometry analysis was performed.

After high pH liquid phase separation and liquid-mass spectrometry, the enzymolyzed peptides of each sample were collected separately on the computer, and a spectral library was established using Spectreonaut Pulsar X software for data analysis. The *t*-test was performed on the repeated values of each group to calculate the fold change and *P value* of each comparison group, and then two-standard screening was performed (fold change = 1.2, *P value* < 0.05). Proteins (fold change > 1.2 or < 5/6, *P value* < 0.05) were considered to be significantly differentially expressed proteins, which are listed and marked with colors.

### Enzyme-linked immunosorbent assay

Large sample verification of the proteins (fold change > 1.2, P value < 0.01) was performed by enzyme-linked immunosorbent assay (ELISA) using commercial kits purchased from RayBiotec, Inc. (Norcross, GA), R&D Systems (Minneapolis, MN), and Abbexa, Inc. (Beijing, China). The experiments were conducted by following the manufacturer’s suggested procedures.

### Statistical analysis

Concentrations of thrombospondin-1 (TSP1), glutathione S-transferase omega-1 (GSTO1), inhibin beta C chain (INHBC), tetranectin (CLEC3B), extracellular matrix protein 1 (ECM-1) and β-hCG in the serum of IP and EP women are presented as the mean ± standard deviation. The t-test was conducted for independent samples of the two groups according to ELISA verification results, and a P value < 0.05 was considered statistically significant. The Hosmer–Lemeshow test was performed to assess the calibration degree of the prediction model. The ROC curve was made by GraphPad Software (San Diego, CA), and the area under the curve (AUC), sensitivity, specificity, positive predictive value and negative predictive value were calculated to evaluate the diagnostic value of EP.

### Institutional Review Board Statement

All subjects gave their informed consent for inclusion before they participated in the study. The study was conducted in accordance with the Declaration of Helsinki, and the protocol was approved by the Ethics Committee of West China Second Hospital of Sichuan University(no.124).

## Supplementary Information

Supplementary Information.

## Data Availability

All datasets generated for this study are available within the article.
