# Supplementary material for: Identification of noninvasive diagnostic biomarkers for ectopic pregnancy using data-independent acquisition (DIA)proteomics: a pilot study
Source: Sci Rep. 2022 Nov 21;12:19992. doi: 10.1038/s41598-022-23374-8 (PMC9678856; doi:10.1038/s41598-022-23374-8)
Supplement: Supplementary file 1 — Supplementary Information. [file 41598_2022_23374_MOESM1_ESM.pdf]

Supplemental Table 1. Detailed information of samples selected for proteomic screening assay

Samples	IP			EP		
	Age, year	Gestational age, day	β-hCG (mIU/mL)	Age, year	Gestational age, day	β-hCG (mIU/mL)
1	35	41	26127.9	30	54	47975.8
2	29	43	53407.4	29	48	8656.5
3	35	56	6293.1	27	69	1086.9
4	28	67	102720.6	37	41	1125.7
5	28	49	43764.6	32	49	35257
